# Quality of life in children with tuberous sclerosis complex: A pediatric cohort study

**DOI:** 10.1111/cns.13473

**Published:** 2020-11-23

**Authors:** Yifeng Ding, Ji Wang, Yuanfeng Zhou, Lifei Yu, Linmei Zhang, Shuizhen Zhou, Yi Wang

**Affiliations:** ^1^ Department of Neurology Children's Hospital of Fudan University Shanghai China

**Keywords:** child, epilepsy, generic core scale, health, PedsQL, TSC

## Abstract

**Aims:**

To evaluate the quality‐of‐life (QOL) impairment and identify the possible risk factors in patients with tuberous sclerosis complex (TSC) in China.

**Methods:**

The parent proxy‐report PedsQL 4.0 Generic Core Scales were administered to 124 caregivers of children with TSC (aged 2‐18 years). For comparison, the survey was also conducted in a demographically group‐matched sample of healthy controls (HCs) (aged 2‐18 years).

**Results:**

A total of 124 children with TSC and 206 HCs were recruited. The mean parent proxy‐report total scale score, physical health summary score, and psychosocial health summary score for children with TSC were 65.0 (SD 19.7), 77.6 (SD 22.9), and 58.0 (SD 21.3), respectively, compared with the HC values of 83.6 (SD 14.3), 87.2 (SD 16.9), and 82.8 (SD 15.9). There were statistically significant differences between the two groups (*P* < .0001). *TSC2* mutation (*P* = .033), epilepsy (*P* = .011), seizure before 2 years old (*P* = .001), course of epilepsy (more than 2 years) (*P* = .001), high reported seizure frequency (more than once a month) (HRSF) (*P* = .007), multiple antiepileptic drugs (≥2) (*P* = .002), intellectual disability (ID) (mild and moderate ID, *P* < .0001, and severe and profound ID, *P* < .0001), and TANDs (*P* < .0001) (ADHD, *P* = .004; agoraphobia, *P* = .007; and social anxiety disorder, *P* < .0001) were closely related to lower QOL scores.

**Conclusion:**

This study is the first large cohort study on QOL in children with TSC in China. The results of the PedsQL 4.0 indicated that the QOL of children with TSC is significantly lower than that of HCs. *TSC2* mutation, epilepsy, early onset, long disease course and HRSF, ID, and TANDs are risk factors for poor QOL.

## Graphical abstract

The quality of life of children with TSC is significantly lower than that of HCs. *TSC2* mutation, epilepsy, early onset, long disease course and high reported seizure frequency (more than once a month), intellectual disability, and tuberous sclerosis–associated neuropsychiatric disorders are risk factors for poor quality of life.

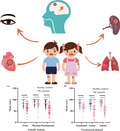

## 1 INTRODUCTION

Tuberous sclerosis complex (TSC, OMIM #191100, #613254), a genetic disease with an autosomal dominant inheritance, affects those who have it across their life span and occurs in approximately 1 in 5000‐10 000 live births.^1^, ^2^, ^3^ In our previous study,^4^ epilepsy affected 85.06 percent of our cohort of 174 patients diagnosed with TSC. Intellectual disability is a primary feature of TSC, affecting 44‐70 percent of patients in population‐based reports. The prevalence of significant behavioral problems among children with TSC ranges from 40 to 90 percent.^5^, ^6^, ^7^ However, only a few studies have assessed the associated total disease burden and quality of life (QOL).

As one of the most widely used health‐related quality‐of‐life measures for patients aged 2‐18 years, the Pediatric Quality of Life Inventory (PedsQL) 4.0 Generic Core Scales are stable, reliable, repeatable, and suitable for general and disease‐specific populations.^8^, ^9^, ^10^, ^11^, ^12^ This cross‐sectional study was designed to (a) investigate the QOL of Chinese patients with TSC (aged 2‐18 years); (b) compare their QOL with that of healthy controls (HCs); and (c) more precisely explore risk factors for lower QOL scores.

## 2 MATERIALS AND METHODS

### 2.1 Subjects

During the inclusion period (June 2019‐December 2019), all children with TSC aged 2‐18 years and their parents received an invitation from Children's Hospital of Fudan University. A diagnosis of TSC was based on the latest diagnostic criteria for TSC.^13^ The only exclusion criterion was an insufficient understanding of the Chinese language or unwillingness to participate in the study. A total of 206 healthy individuals aged 2‐18 years were recruited from schools to create an age‐ and sex‐matched control group.

This study was subject to approval by the ethics committee of the Children's Hospital of Fudan University (2018‐No.26). Written informed consent and verbal assent were provided by all parents and participants (aged ≥ 12 years).

### 2.2 Data collection

#### 2.2.1 Standardized questionnaire

Demographic characteristics (sex, birth date, ethnicity, years of education of the parents, monthly household income, and urban/rural residence) and clinical data (age at onset of epilepsy, course of epilepsy, antiepileptic drugs, seizure frequency, family history, multisystem clinical manifestations of TSC, etc) were assessed by a standard structured questionnaire.

#### 2.2.2 PedsQL 4.0 generic core scales

The Mandarin Chinese version of the PedsQL 4.0 parent proxy report was used to assess the QOL of Chinese children with TSC.

There are four age‐group scales specific to children aged 2‐4, 5‐7, 8‐12, and 13‐18 years, and these are used to assess parents' perceptions of the QOL of children with TSC. The 21‐item (2‐4 years)/23‐item (5‐18 years) parent proxy‐report PedsQL 4.0 consists of the following 4 domains: (a) physical functioning (8 items), (b) emotional functioning (5 items), (c) social functioning (5 items), and (d) school functioning (5 items). The reliability and validity of the Chinese translations of the PedsQL 4.0 have been demonstrated in Chinese children.^14^, ^15^, ^16^, ^17^, ^18^, ^19^ Emotional, school, and social functioning can also be united into a psychosocial health domain. Each item is rated using a 5‐point response scale from 0 = "never is a problem" to 4 = "almost always a problem." The weights of the items are equal, the score is reversed, and the linear conversion is 0‐100 points (0 = 100, 1 = 75, 2 = 50, 3 = 25, 4 = 0). Thus, the higher the function score is, the better the QOL is. Face‐to‐face interviews were conducted by trained neurologists to collect the data.

#### 2.2.3 Statistical analysis

The statistical analyses were conducted with JMP Pro software, version 14.3.0 (SAS Institute, USA). The Shapiro‐Wilk normality test was used to test the normality of the data distribution. The unpaired *t* test for continuous data and the chi‐square test or Fisher's exact test for categorical data were used to test for sociodemographic differences between TSC patients and HCs. The unpaired *t* test was used for the comparison of the parent proxy‐report PedsQL 4.0 scores between the two groups. ANOVA F tests were used to compare the PedsQL 4.0 subscores among all of the age‐groups and different genotype groups (*TSC1*, *TSC2,* and NMI). We used multivariate linear regression models to identify risk factors for low QOL scores. Some potential confounding factors were adjusted in Models 1b, 2b, and 3b, such as sex, parents' years of education, monthly household income, and residence. A *P*‐value < .05 was considered significant.

## 3 RESULTS

### 3.1 Characteristics of the study population

Altogether, 124 children with TSC (median age ± standard deviation: 8.5 ± 3.7 years) who met the inclusion criteria were enrolled in the study and returned a questionnaire completed by their parents: 61 (49.2%) were boys and 63 (50.8%) were girls. A total of 206 gender‐matched (male: female = 109:97) and age‐matched (9.3 ± 4.1 years) HCs were recruited (Table 1).

**Table 1:** Sociodemographic comparison of TSC patients and healthy controls

Sociodemographic features	TSC	HCs	*P‐*value
N	124	206	
Male	61 (49.2)	109 (52.9)	.57
Age at interview (y)	8.5 ± 3.7	9.3 ± 4.1	.08
Age subgroups (y)			.99
2‐4	16 (12.9)	27 (13.1)	
5‐7	34 (27.4)	57 (27.6)	
8‐18	74 (59.7)	122 (59.3)	
Father's years of education (y)			.78
≤9	43 (34.7)	65 (31.6)	
9‐12	28 (22.6)	45 (21.8)	
>12	53 (42.7)	96 (46.6)	
Mothers’ years of education (y)			.88
≤9	46 (37.1)	71 (34.5)	
9‐12	24 (19.4)	40 (19.4)	
>12	54 (43.6)	95 (46.1)	
Monthly household income (RMB)			.39
<5000	21 (16.9)	26 (12.6)	
5000‐10 000	63 (50.8)	119 (57.8)	
>10 000	40 (32.3)	61 (29.6)	
Residence			.11
Suburban or rural	62 (50.0)	122 (59.2)	
Urban	62 (50.0)	84 (40.8)	

Note

TSC, TSC group; HCs, healthy controls

Data are presented as the median ± SD and n (%).

ANOVA F tests were used for continuous variables and chi‐square tests or Fisher's exact tests for categorical data.

All 124 patients had undergone genetic testing, and the results showed that the ratio of *TSC1*, *TSC2,* and NMI (no mutation identified) was 34:76:14. According to the DSM‐5 criteria, 81 (65.3%) patients out of 124 presented with intellectual disability (ID), with mild ID (IQ 51‐70) and moderate ID (IQ 36‐50) accounting for 33.9% (42/124) and severe ID (IQ 20‐35) and profound ID (IQ < 20) for 31.5% (39/124). Comorbid epilepsy diagnoses were found in 84.7% (105/124) of the TSC patients. Two or more seizure types were detected in 65.7% (69/105) of all patients with epilepsy. The most common type of epilepsy was focal motor epilepsy (75/105; 71.4%). As the characteristic seizure type, infantile spasms were diagnosed in 44.8% (47/105) of patients with epilepsy. Of the 124 patients with neuroimaging results available, 91.6% (114/124) had tubers or cortical dysplasias, 90.3% (112/124) had subependymal nodules (SENs), and 4.0% (5/124) had subependymal giant cell astrocytoma (SEGA). A total of 15 tuberous sclerosis–associated neuropsychiatric disorders (TANDs) were diagnosed in 95 patients aged 6‐16 years using the Mini International Neuropsychiatric Interview for patients. The incidence rates from low to high were as follows: conduct disorder (1.1%), posttraumatic stress disorder (3.2%), obsessive‐compulsive disorder (6.3%; 6/95), dysthymia (4.2%), major depressive episode (6.3%), suicide (6.3%), oppositional defiant disorder (7.4%), separation anxiety disorder (10.5%), tic disorder (15.8%), agoraphobia (16.8%), (mild) manic episodes (22.1%), specific phobia (26.3%), panic disorder (26.3%), social anxiety disorder (41.1%), and attention‐deficit/hyperactivity disorder (ADHD; 51.6%) (https://www.researchsquare.com/article/rs‐32617/v1).

### 3.2 Comparison between the TSC group and healthy controls

**Figure 1:**
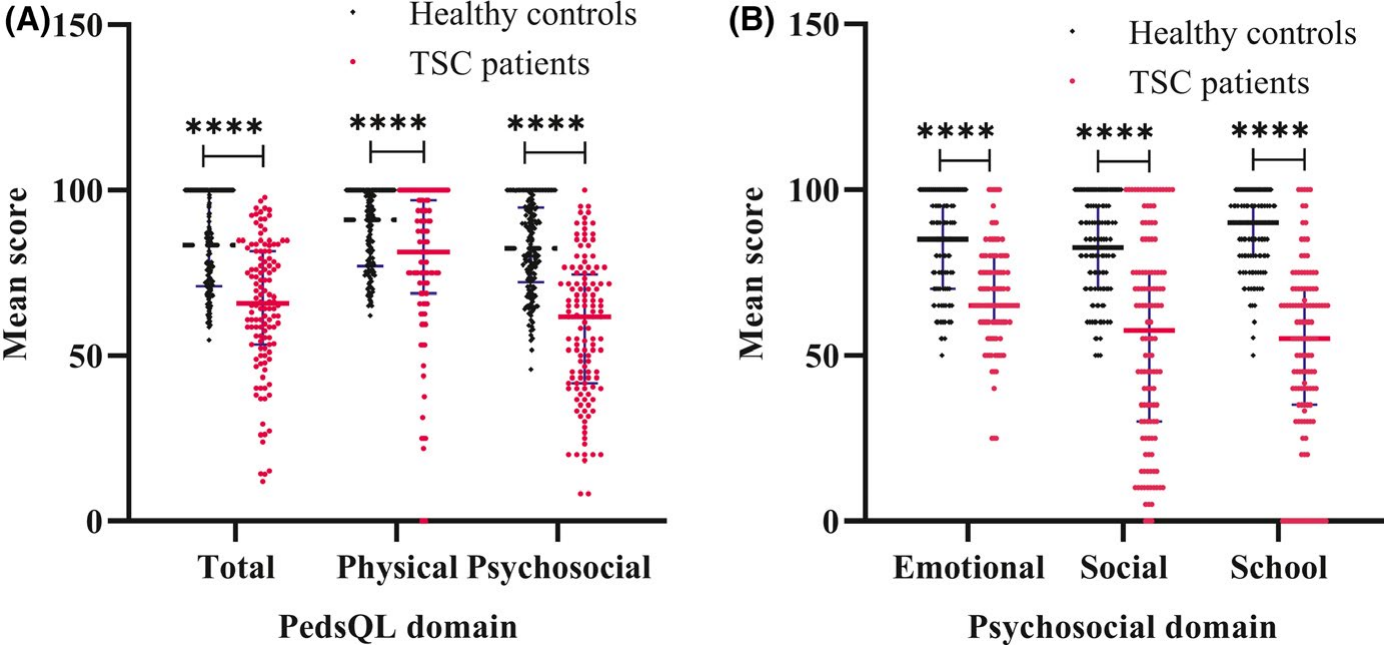
Comparison of parent proxy‐report PedsQL 4.0 scores between children with TSC and healthy controls. Data are presented as the median ± SD; ****P < .0001. Unpaired *t* tests or unpaired *t* tests with Welch's correction were used

The mean total scale score, physical health summary score, and psychosocial health summary score for children with TSC were 65.0 (SD 19.7), 77.6 (SD 22.9), and 58.0 (SD 21.3), respectively, compared with the HC values of 83.6 (SD 14.3), 87.2 (SD 16.9), and 82.8 (SD 15.9). There were statistically significant differences between the two groups (*P* < .0001) (Figure 1A). The QOL of children with TSC was significantly worse than that of HCs. As shown in Figure 1B, there were also significant differences in the social, emotional, and school functioning scale scores between the two groups.

There were no significant differences among any of the age‐groups for any of the QOL domains (*P* > .05). On average, the parent‐reported psychosocial health scores were significantly lower than the physical health scores (*P* < .0001). For the psychosocial domains, compared with the NMI group, the *TSC1/TSC2* group had significantly lower scores (*P *= .016) (emotional functioning, *P* = .027; school functioning, *P* = .028; social functioning, *P* = .037).

### 3.3 Analysis of risk factors for lower QOL scores

Multiple linear regression models adjusted for potential confounders were used to explore the associations between QOL scores and clinical manifestations of TSC (including TANDs).

As shown in Table 2, *TSC2* mutation (*P* = .033), epilepsy (*P* = .011), seizure before 2 years old (*P* = .001), course of epilepsy (more than two years) (*P* = .001), HRSF (more than once per month) (*P* = .007), multiple antiepileptic drugs (≥2) (*P* = .002), ID (mild and moderate ID, *P* < .0001, and severe and profound ID, *P* < .0001), and TANDs (*P* < .0001) (ADHD, *P* = .004; agoraphobia, *P* = .007; and social anxiety disorder, *P* < .0001) were closely related to lower QOL scores after adjusting for sex (male/female), mother's years of education (≤9/9‐12/>12), father's years of education (≤9/9‐12/>12), monthly household income (RMB) (<5000/5000‐10 000/>10 000), and residence (suburban or rural/urban) in Model 1b.

**Table 2:** The associations between total scale scores and clinical manifestations of TSC (including TANDs)

Predictor	n (%)	Mean parent proxy‐report total scale score			
		Model 1a β1 (95% CI)	*P‐*value	Model 1b β2 (95% CI)	*P‐*value
Gene					
NMI	14 (11.3)	Ref.	/	Ref.	/
*TSC1*	34 (27.4)	−8.05 (−20.30, 4.20)	.200	−7.79 (−20.87, 5.30)	.241
*TSC2*	76 (61.3)	−11.55 (−22.77, −0.32)	.040^*^	−12.91 (−24.79, −1.03)	.033^*^
Epilepsy					
No	19 (15.3)	Ref.	/	Ref.	/
Yes	105 (84.7)	−12.86 (−22.33, −3.38)	.008^*^	−13.04 (−23.10, −2.99)	.011^*^
Seizure before 2 y old					
No	38 (36.2)	Ref.	/	Ref.	/
Yes	67 (63.8)	−13.28 (−21.14, −5.42)	.001^*^	−14.00 (−22.33, −5.68)	.001^*^
Course of epilepsy (≥2 y)					
No	38 (36.2)	Ref.	/	Ref.	/
Yes	67 (63.8)	−13.77 (−21.49, −6.05)	.001^*^	−13.79 (−22.02, −5.56)	.001^*^
Multiple antiepileptic drugs (≥2)					
No	60 (49.6)	Ref.	/	Ref.	/
Yes	61 (50.4)	−12.09 (−18.94, −5.24)	.001^*^	−11.81 (−19.10, −4.52)	.002^*^
HRSF					
No	65 (61.9)	Ref.	/	Ref.	/
Yes	40 (38.1)	−11.05 (−18.85, −3.26)	.006^*^	−11.33 (−19.42, −3.23)	.007^*^
IQ					
Normal	43 (34.7)	Ref.	/	Ref.	/
Mild and moderate ID	42 (33.9)	−14.93 (−21.06, −8.81)	<.0001^*^	−16.40 (−22.76, −10.05)	<.0001^*^
Severe and profound ID	39 (31.5)	−33.51 (−39.75, −27.27)	<.0001^*^	−35.77 (−42.29, −29.25)	<.0001^*^
TANDs					
No	16 (16.8)	Ref.	/	Ref.	/
Yes	95 (83.2)	−21.89 (−31.20, −12.58)	<.0001^*^	−21.75 (−31.59, −11.93)	<.0001^*^
ADHD					
No	46 (48.4)	Ref.	/	Ref.	/
Yes	49 (51.6)	−10.58 (−18.02, −3.15)	.001^*^	−11.69 (−19.57, −3.80)	.004^*^
Social anxiety disorder					
No	57 (60)	Ref.	/	Ref.	/
Yes	38 (40)	−14.64 (−21.95, −7.34)	.001^*^	−15.00 (−22.58, −7.43)	<.0001^*^
Agoraphobia					
No	80 (84.2)	Ref.	/	Ref.	/
Yes	15 (15.8)	−14.93 (−25.09, −4.76)	.004^*^	−14.73 (−25.28, −4.17)	.007^*^

Note

Abbreviations: ADHD, attention‐deficit/hyperactivity disorder; AED, antiepileptic drugs; HRSF, high reported seizure frequency (more than once per month); ID, intellectual disability; IQ, intelligence quotient; NMI, no mutation identified; TANDs, tuberous sclerosis–associated neuropsychiatric disorders.

Data are presented as n (%).

Model 1a: unadjusted

Model 1b: adjusted for gender (male/female), mother's years of education (≤9/9‐12/>12), father's years of education (≤9/9‐12/>12), monthly household income (RMB) (<5000/5000‐10 000/>10 000), and residence (suburban or rural/urban).

As shown in Table 3, high reported HSF (*P* = .015), multiple antiepileptic drugs (≥2) (*P* < .0001), ID (mild and moderate ID, *P* = .001, and severe and profound ID, *P* < .0001), and TANDs (*P* < .0001) (social anxiety disorder, *P* = .002) were closely related to lower physical health summary scores after adjusting for sex (male/female), mother's years of education, father's years of education, monthly household income, and residence in Model 2b.

**Table 3:** The associations between the physical health summary scores and clinical manifestations of TSC (including TANDs)

Predictor	n (%)	Mean parent proxy‐report physical scale scores			
		Model 2a β1 (95% CI)	*P‐*value	Model 2b β2 (95% CI)	*P‐*value
Gene					
NMI	14 (11.3)	Ref.	/	Ref.	/
*TSC1*	34 (27.4)	−0.25 (−14.61, 14.11)	.973	−0.99 (−16.15, 14.17)	.897
*TSC2*	76 (61.3)	−7.35 (20.50, 5.80)	.271	−9.71 (−23.47, 4.06)	.165
Epilepsy					
No	19 (15.3)	Ref.	/	Ref.	/
Yes	105 (84.7)	−9.59 (−20.82, 1.64)	.093	−9.74 (−21.53, 2.05)	.104
Seizure before 2 y old					
No	38 (36.2)	Ref.	/	Ref.	/
Yes	67 (63.8)	−9.42 (−19.06, 0.22)	.001^*^	−9.31 (−19.45, 0.83)	.071
Course of epilepsy (≥2 y)					
No	38 (36.2)	Ref.	/	Ref.	/
Yes	67 (63.8)	−9.74 (−19.24, −0.24)	.045^*^	−8.86 (−18.89, 1.18)	.083
Multiple antiepileptic drugs (≥2)					
No	60 (49.6)	Ref.	/	Ref.	/
Yes	61 (50.4)	−16.05 (−23.86, −9.24)	<.0001^*^	−15.36 (−23.60, −7.12)	<.0001^*^
HRSF					
No	65 (61.9)	Ref.	/	Ref.	/
Yes	40 (38.1)	−10.99 (−20.34, −1.64)	.022^*^	−11.99 (−21.55, −2.43)	.015^*^
IQ					
Normal	43 (34.7)	Ref.	/	Ref.	/
Mild and moderate ID	42 (33.9)	−13.54 (−22.21, −4.87)	.002^*^	−15.06 (−24.00, −6.13)	.001^*^
Severe and profound ID	39 (31.5)	−27.39 (−36.22, −18.55)	<.0001^*^	−30.21 (−39.38, −21.04)	<.0001^*^
TANDs					
No	16 (16.8)	Ref.	/	Ref.	/
Yes	95 (83.2)	−21.89 (−31.20, −12.58)	<.0001^*^	−21.75 (−31.59, −11.93)	<.0001^*^
ADHD					
No	46 (48.4)	Ref.	/	Ref.	/
Yes	49 (51.6)	−8.08 (−16.61, −0.46)	.063	−8.98 (−18.08, 0.12)	.053
Social anxiety disorder					
No	57 (60)	Ref.	/	Ref.	/
Yes	38 (40)	−12.83 (−21.30, −4.35)	.003^*^	−13.83 (−22.59, −5.06)	.002^*^
Agoraphobia					
No	80 (84.2)	Ref.	/	Ref.	/
Yes	15 (15.8)	−9.71 (−21.46, 2.03)	.104	−9.27 (−21.49, 2.96)	.136

Note

Abbreviations: ADHD, attention‐deficit/hyperactivity disorder; AED, antiepileptic drugs; HRSF, high reported seizure frequency (more than once per month); ID, intellectual disability; IQ, intelligence quotient; NMI, no mutation identified; TANDs, tuberous sclerosis–associated neuropsychiatric disorders.

Data are presented as n (%).

Model 2a: unadjusted

Model 2b: adjusted for gender (male/female), mother's years of education (≤9/9‐12/>12), father's years of education (≤9/9‐12/>12), monthly household income (RMB) (<5000/5000‐10 000/>10 000), and residence (suburban or rural/urban).

As shown in Table 4, *TSC2* mutation (*P* = .031), epilepsy (*P* = .007), seizure before 2 years old (*P* = .001), course of epilepsy (more than 2 years) (*P* < .0001), HRSF (*P* = .012), multiple antiepileptic drugs (≥2) (*P* = .012), ID (mild and moderate ID, *P* < .0001, and severe and profound ID, *P* < .0001), and TANDs (*P* < .0001) (ADHD, *P* = .004; agoraphobia, *P* = .003; and social anxiety disorder, *P = *.001) were closely related to lower psychosocial health summary scores after adjusting for sex (male/female), mothers' years of education, father's years of education, monthly household income, and residence in Model 3b.

**Table 4:** The associations between the psychosocial health summary scores and clinical manifestations of TSC (including TANDs)

Predictor	n (%)	Mean parent proxy‐report psychosocial scale score			
		Model 3a β1 (95% CI)	*P‐*value	Model 3b β2 (95% CI)	*P‐*value
Gene					
NMI	14 (11.3)	Ref.	/	Ref.	/
*TSC1*	*34 (27.4)*	−*11.96 (−25.21, 1.29)*	*.076*	−*11.19 (−25.40, 3.02)*	*.121*
*TSC2*	76 (61.3)	−13.30 (25.43, −1.16)	.032^*^	−14.23 (−27.13, −1.33)	.031^*^
Epilepsy					
No	19 (15.3)	Ref.	/	Ref.	/
Yes	105 (84.7)	−14.88 (−25.09, −4.66)	.005^*^	−15.13 (−25.98, −4.28)	.007^*^
Seizure before 2 y old					
No	38 (36.2)	Ref.	/	Ref.	/
Yes	67 (63.8)	−14.78 (−22.23, −6.33)	.001^*^	−16.10 (−25.04, −7.15)	.001^*^
Course of epilepsy (≥2 y)					
No	38 (36.2)	Ref.	/	Ref.	/
Yes	67 (63.8)	−15.39 (−23.68, −7.10)	<.0001^*^	−16.04 (−24.87, −7.21)	<.0001^*^
Multiple antiepileptic drugs (≥2 AEDs)					
No	60 (49.6)	Ref.	/	Ref.	/
Yes	61 (50.4)	−10.42 (−17.95, −2.89)	.007^*^	−10.28 (−18.30, −2.27)	.012^*^
HRSF					
No	65 (61.9)	Ref.	/	Ref.	/
Yes	40 (38.1)	−11.44 (−19.88, −3.00)	.008^*^	−11.35 (−20.16, −2.53)	.012^*^
IQ					
Normal	43 (34.7)	Ref.	/	Ref.	/
Mild and moderate ID	42 (33.9)	−15.70 (−22.16, −9.24)	<.0001^*^	−17.17 (−23.95, −10.39)	<.0001^*^
Severe and profound ID	39 (31.5)	−37.22 (−43.81, −30.63)	<.0001^*^	−39.31 (−46.27, −32.35)	<.0001^*^
TANDs					
No	16 (16.8)	Ref.	/	Ref.	/
Yes	95 (83.2)	−24.90 (−35.02, −14.78)	<.0001^*^	−24.46 (−35.13, −13.80)	<.0001^*^
ADHD					
No	46 (48.4)	Ref.	/	Ref.	/
Yes	49 (51.6)	−11.76 (−19.90, −3.61)	.005^*^	−12.88 (−21.49, −4.27)	.004^*^
Social anxiety disorder					
No	57 (60)	Ref.	/	Ref.	/
Yes	38 (40)	−15.24 (−23.32, −7.17)	<.0001^*^	−15.24 (−23.62, −6.85)	.001^*^
Agoraphobia					
No	80 (84.2)	Ref.	/	Ref.	/
Yes	15 (15.8)	−17.44 (−28.52, −6.37)	.002^*^	−17.38 (−28.83, −5.93)	.003^*^

Note

Abbreviations: ADHD, attention‐deficit/hyperactivity disorder; AED, antiepileptic drugs; HRSF, high reported seizure frequency (more than once per month); ID, intellectual disability; IQ, intelligence quotient; NMI, no mutation identified; TANDs, tuberous sclerosis–associated neuropsychiatric disorders.

Data are presented as n (%).

Model 3a: unadjusted.

Model 3b: adjusted for gender (male/female), mother's years of education (≤9/9‐12/>12), father's years of education (≤9/9‐12/>12), monthly household income (RMB) (<5000/5000‐10 000/>10 000), and residence (suburban or rural/urban).

## 4 DISCUSSION

As far as we know, this study was the first cohort study of QOL in children with TSC in China. A total of 65.3% of patients presented with ID, which is why we chose the parent proxy version. The mean total scale score, psychosocial score, and physical health score for the children with TSC were significantly lower than those in the healthy population. QOL in patients with TSC was significantly compromised compared with that in the healthy group, as might be expected.

Notably, the psychosocial health scores were significantly lower than the physical health scores, which was consistent with previous studies.^20^, ^21^ As in previous studies, TANDs can exist at all ages.^5^ The lifetime cumulative incidence of TANDs is approximately 90%.^22^ The incidence of neuropsychiatric comorbidities was significantly higher in TSC patients than in HCs.^7^, ^23^ Our findings indicate that neuropsychiatric disorders seriously affect patients' QOL and require careful attention from neurologists. However, our results are quite different from those of previous studies: There were no significant differences in any of the QOL domains by age.^20^ It was first found that the *TSC1/TSC2* group reported significantly worse QOL. Similar to previous studies, *TSC1/TSC2* pathogenic variants could generally cause a more severe clinical phenotype.^4^, ^24^, ^25^, ^26^, ^27^, ^28^

Patient QOL is affected by the interaction of all phenotypes.^29^, ^30^ This study is probably the most comprehensive study on risk factors for QOL to be conducted thus far.^20^, ^21^, ^31^ Multiple linear regression models were adopted to identify possible risk factors independently related to the low proxy‐report PedsQL scores. Simple linear regression revealed that *TSC2* mutation, epilepsy, seizure before 2 years old, course of epilepsy, HRSF, multiple antiepileptic drugs, ID, and TANDs could be associated with poor QOL. After adjusting for all of the possible confounding factors (sex, maternal education, paternal education, family income, and residence), HRSF, use of a greater number of antiepileptic drugs (≥2), ID, and TANDs were significant independent risk factors for all QOL domains. In addition, *TSC2* mutation, epilepsy, seizure before 2 years old, and course of epilepsy ≥ 2 years were also related to lower psychosocial health scores, but not to physical health scores. In this study, neither multisystem clinical manifestations of TSC (brain, heart, skin, eyes, kidney, lung, and liver) nor the *TSC1/TSC2* gene mutation type was associated with poor QOL. In agreement with previous research, we consider epilepsy an important factor affecting the QOL of children with TSC. The earlier the age of onset of epilepsy, the more antiepileptic drugs needed for refractory epilepsy, and the longer the course of epilepsy, the greater the impact on QOL.^31^ Treatments for epilepsy in early life will help to improve QOL in epilepsy care.^32^, ^33^

In view of the diversity of clinical manifestations in TSC patients, the burden of disease is highly variable,^34^, ^35^ and the impact on QOL is also different. Research on risk factors will help to improve the QOL of children with TSC. Follow‐up of these children with TSC is ongoing, with additional future focus on QOL solutions for this population. Early screening, timely diagnosis, and correct intervention for risk factors are essential to improving their QOL. Psychosocial‐based interventions also represent a major opportunity to enhance QOL.

This study has several limitations, including those inherent to a single‐center cross‐sectional study design. Further prospective multicenter studies are needed to validate these results. Considering the relatively high proportion of ID, parent proxy reports were used. Although the selected reporters were close caregivers of the child, there may still be certain deviations. The MINI‐KID scale cannot be used to screen and diagnose ASD, and the age‐group is limited to those aged 6‐16 years old. In our study, epilepsy and TAND are important risk factors that affect quality of life. To reduce bias, future studies might need to compare the quality of life of children with chronic neurological and non‐neurological diseases, such as epilepsy, diabetes, narcolepsy, and psychosis, and assess the impact of sleep disorders and rehabilitation on quality of life.^8^, ^36^, ^37^

## 5 CONCLUSIONS

This study was the first large cohort study of QOL in children with TSC in China. The results indicated that the QOL of children with TSC is significantly lower than that of HCs. *TSC2* mutation, epilepsy, seizure before 2 years old, course of epilepsy (more than 2 years), HRSF, multiple antiepileptic drugs (≥2), ID, and TANDs were closely related to poor QOL.

## CONFLICT OF INTEREST

The authors declare no conflict of interest.

## Data Availability

The datasets used during and/or analyzed during the current study are available from the corresponding author on request.
